# Editorial: The Biological and Clinical Aspects of HLA-G, Volume II

**DOI:** 10.3389/fimmu.2022.959706

**Published:** 2022-06-28

**Authors:** Joel LeMaoult, Wei-Hua Yan

**Affiliations:** ^1^ Commissariat ã l’Energie Atomique et aux Energies Alternatives, DRF, Francois Jacob Institute of Biology, Hemato-Immunology Research Department, Saint-Louis Hospital, Paris, France; ^2^ U976 HIPI Unit, IRSL, Université Paris, Paris, France; ^3^ Medical Research Center, Taizhou Hospital of Zhejiang Province, Wenzhou Medical University, Linhai, China; ^4^ Key Laboratory of Minimally Invasive Techniques & Rapid Rehabilitation of Digestive System Tumor of Zhejiang Province, Taizhou Hospital of Zhejiang Province, Linhai, China

**Keywords:** HLA-G, receptor, immune regulation, cancer, autoimmune, infection, reproduction

Human leukocyte antigen G (HLA-G), a non-classical class HLA-I antigen, is an important immune regulator in homeostasis and disease progression. Biological and clinical significance of HLA-G expression and genetic variation of *HLA-G* in different setting have been extensively investigated since its discovery.

In this Research Topic “The Biological and Clinical Aspects of HLA-G volume II”, fifteen in-depth reviews, perspectives and original research articles on different aspects of HLA-G were published, which focused on HLA-G and its receptors in cancer, autoimmune and infectious diseases, skin, allergy and reproduction immunity.

Multiple HLA-G isoforms generated by primary transcript alternative splicing were identified, such as α1 domain-containing (HLA-G1~HLA-G7) and α1 domain-deleted isoforms. It is expected that all HLA-G spliceforms are immune inhibitory, but given the extent of their structural diversity, it is also expected that differences in their biological functions exist. The immune checkpoint functions of HLA-G were shown to be either beneficial or deleterious, depending on the biological context. Indeed, HLA-G was shown to promote fetal-maternal immune tolerance, limit inflammation, and prolong transplanted grafts acceptance. Inversely, it was also shown to impair host immune responses against virus-infected cells and malignant cells, and enhance the capability of these abnormal cells to escape immune clearance. The HLA-G receptors ILT2/LILRB1, ILT4/LILRB2, and KIR2DL4 were shown to bind α1 domain-containing isoforms. KIR2DL4 recognizes HLA-G through its α1-α2 domains, while ILT2 and ILT4 primarily recognize HLA-G α3 domain of B2M-associated isoforms (ILT2) or B2M-free isoforms (ILT4). Functionally, such interactions lead to immune inhibition. In this regard, an increasing number of clinical trials on HLA-G and/or ILTs targeted immunotherapy for solid cancers have been started. Binding of ILT2 and ILT4 receptors to α1-deleted isoforms is unknown. However, α1 domain-deleted isoform has been recently found to be with stimulatory property, raising questions regarding the receptors involved and warranting more work to explore HLA-G functional complexity.

Fetal trophoblasts are key players in fetal-maternal interaction during pregnancy. They constitutively express HLA-G, especially extravillous trophoblast cells, and this expression is required for maternal-fetal tolerance and successful implantation. Eikmans et al. present a method to culture trophoblasts from first-term placentas, and differentiate them into extravillous trophoblasts expressing HLA-G. This is a unique model to study naturally expressed HLA-G and conduct research on fetal-maternal immune cross-talk.

Neo-expression of HLA-G is commonly observed among almost all types of cancers. Through engagement with its receptors, HLA-G expression by cancer cells exerts an immune checkpoint function and induces immune suppression by inhibiting the functions of immune effectors, and by induction the proliferation of immune regulatory cells. Thus, there is a growing interest in blocking HLA-G:HLA-G-Receptor signaling blockade in cancer immunotherapy. Martı´n-Villa et al. provide an exhaustive review on the genetic variation of the *HLA-G* gene, on the mechanisms of HLA-G-mediated immune suppression and its relevance in cancer and autoimmune diseases. Lin and Yan review the possible HLA-G:ILTs-targeting immunotherapies for solid cancers, while also highlighting the major challenges that remain to be addressed in this context, including the diversity of HLA-G isoforms, theand intra- and inter-tumor heterogeneity of HLA-G expression. This should be taken into consideration when developping clinical applications. In this regard, HLA-G/KIR2DL4 signaling can impair ADCC of NK cells with the treatment of Her-2 antibody trastuzumab. Zheng et al. discuss the HLA-G/KIR2DL4 signaling in breast cancer microenvironment and its mechanisms and effects in breast cancer immunotherapy, providing a new light on significance of HLA-G/KIR2DL4 in cancer cell immune evasion.

Genetic variation of *HLA-G* has been shown to be related to HLA-G expression levels and predisposition to certain types of diseases. In this context, Scavuzzi et al. present the “MHC Cusp Theory” which postulates that in addition to its main role in antigen presentation, the MHC codes for allele-specific molecules that act as ligands in a conformationally-conserved cusp-like fold, which upon interaction with cognate receptors can trigger MHC-associated diseases. Based on the structural and functional features shares among HLA-G and other HLA antigens, and on how specific HLA-G allelic molecules differently affect NK functions, the authors postulate that HLA-G isoforms and allelic products might also contain cusp domain-binding sites. Piekarska et al. investigate association of haplotypes and diplotypes containing single nucleotide polymorphisms rs1632947: c.-964G>A, rs1233334: c.-725G>C/T in *HLA-G* promoter region, and rs371194629: c.∗65_∗66insATTTGTTCATGCCT in *HLA-G* 3’ untranslated region (UTR) with levels of soluble HLA-G in semen and with male fertility status. They show that the G-C-ins haplotype is the most unfavorable one for male fertility, which is related to the lowest soluble HLA-G in semen. Rebmann et al. evaluate the relationships between variants in *HLA-G* UTR and disease outcome of patients with locally advanced, non-metastastic breast cancer. Their findings revealed that the UTR-1 or UTR-2 haplotypes are indicators for a better prognosis in term of a complete response to neoadjuvant chemotherapy or progression-free survival, while UTR-4 is a predictor for an inferior overall survival. Jasinski-Bergner et al. review the expression and clinical relevance of HLA-G expression in urological tumors, HLA-G use for diagnostic purposes, as prognostic biomarker, as a monitoring tool for immunotherapy, and as a therapeutic target in urological tumors. Li et al. highlight the significance of the detection of HLA-G molecule derived from different sources such as malignant lesions, peripheral circulating and body fluids, and extracellular vesicles for diagnosis and prognosis among cancer patients. Also, the authors review the limitations of the current HLA-G detection methods and offer opinions on how to standardize HLA-G detection methods.

In the context of inflammatory diseases, Negrini et al. show that increased peripheral soluble HLA-G levels and/or immune cell surface membrane-bound HLA-G molecule can be commonly observed in allergic diseases. In atopic dermatitis, HLA-G expression is found in papillary dermis and infiltrating immune cells such as T cells or Langerhans cells. In skin immunity and tolerance, Mestrallet et al. review the roles of epidermal keratinocytes in skin homeostasis and regeneration. They also highlight the therapeutic potential HLA-G expression modulation in keratinocytes to generate bioengineered universal donor cell sources for skin replacement. Mestrallet et al. further report that HLA-G is overexpressed in CD49f immature keratinocyte precursors with immunosuppressive properties, which can inhibit CD4+ T cell proliferation. DC-10 cells are a subset of DCs involved in IL-10-mediated tolerance. DC-10 are also characterized by a natural HLA-G expression. In patients with Type 1 diabetes (T1D), Amodio et al. reveal that low peripheral blood frequency of tolerogenic DC-10 during disease development is paralleled with the increased proportion of pro-inflammatory cDC2 cells, and that DC-10 impaired CD83 expression is associated with risk of developing T1D.

Induction of HLA-G expression by infected cells is known to be an immune eszcape trategy for viruses. Jasinski-Bergner et al. elaborate mechanisms involved in the induction of HLA-G expression upon various virus infection; and Lin and Yan, review the immunopathological aspects of HLA-G:HLA-G-receptor signalling in SARS-CoV-2 infection, which could provide a better understanding of COVID-19 disease progression and identify potential immunointerventions to counteract SARS-CoV-2 infection.

The field of HLA-G research is fast expanding. With multiple application fields, multiple pathological contexts, biological aspects, and a growing number of new unanswered questions to be investigated, it may be difficult for newcomers to find their mark. We hope the readers of this second Research Topic on HLA-G that focuses on the current advances in the biological and clinical aspects of HLA-G, will find useful information, opinions and perspectives that will help them advance the field. We deeply appreciate all contributions from authors, reviewers and editors alike.

## Author Contributions

All authors listed have made a substantial, direct, and intellectual contribution to the work and approved it for publication.

## Funding

This work was supported by grants from Science Technology Project of Zhejiang Province, China (2020R52060).

## Conflict of Interest

The authors declare that the research was conducted in the absence of any commercial or financial relationships that could be construed as potential conflict of interest.

## Publisher’s Note

All claims expressed in this article are solely those of the authors and do not necessarily represent those of their affiliated organizations, or those of the publisher, the editors and the reviewers. Any product that may be evaluated in this article, or claim that may be made by its manufacturer, is not guaranteed or endorsed by the publisher.

